# Synthesis of Phosphorus-Modified Magnetic Chitosan and Its Application for Cr(VI) Removal from Aqueous Solution

**DOI:** 10.3390/ma18215019

**Published:** 2025-11-04

**Authors:** Hong Wang, Yiran Luo, Qing Hu, Anyuan Cao, Longzhen Ding, Shengbin Xu

**Affiliations:** 1 School of Materials Science and Engineering, Peking University, Beijing 100871, China; wangh6@mail.sustech.edu.cn; 2 School of Environmental Science and Engineering, Southern University of Science and Technology, Shenzhen 518055, China; tushoulalala@163.com (Y.L.); huq@sustech.edu.cn (Q.H.); dinglz@sustech.edu.cn (L.D.); xusb@mail.sustech.edu.cn (S.X.)

**Keywords:** phosphorus-modified magnetic chitosan, hexavalent chromium, adsorption, removal

## Abstract

Traditional Fe-based materials are limited for Cr(VI) remediation due to low reactivity, oxidation, and aggregation. Although chitosan coatings improve stability, they hinder efficient liquid-solid separation. To overcome this, a novel phosphorus-modified magnetic chitosan adsorbent (PCC/Fe_3_O_4_) was synthesized using Fe_3_O_4_ as the core and tetrakis hydroxymethyl phosphonium sulfate (THPS) as a cross-linking agent. The composite exhibited a high surface area (20.67 m^2^/g) and superparamagnetism, enabling easy magnetic recovery. PCC/Fe_3_O_4_ demonstrated superior Cr(VI) removal capabilities compared to unmodified chitosan and raw Fe_3_O_4_, achieving a saturated adsorption capacity of 23.6 mg/g under the selected conditions (pH 6, initial Cr(VI) concentration of 1 mg/L), which were chosen to balance adsorption efficiency, adsorbent stability, and environmental relevance. The main removal mechanism includes electrostatic attraction, redox reaction, and ligand exchange. PCC/Fe_3_O_4_ maintained 86% efficiency after 5 d aging and >90% efficiency after five cycles, demonstrating excellent stability and reusability and strong potential for practical environmental remediation.

## 1. Introduction

Chromium (Cr) contamination at industrial sites, particularly those associated with metal mining and leather tanning, has become a significant environmental concern [1,2]. In contaminated groundwater, Cr primarily exists in oxidation states of Cr(VI), which exhibits high mobility, bioavailability, and toxicity—exceeding that of Cr(III) by more than 1000-fold [3,4,5]. Therefore, conventional remediation strategies focus on introducing reactive materials to reduce Cr(VI) to the less toxic and immobile Cr(III) form.

In recent years, Fe-based materials have gained considerable attention for Cr(VI) remediation due to their ability to release reductive Fe(II) species [6]. However, their practical application is hindered by inherent limitations, including low surface reactivity, susceptibility to oxidation, and aggregation tendencies, which impair their transport and reactivity in contaminated aquifer [7,8]. To address these challenges, recent studies have explored surface modifications and coatings to enhance Fe stability and prevent aggregation [9,10]. Stabilizing agents such as biochar, carboxymethyl cellulose, alumina, silica, and kaolin have been investigated [11,12,13,14,15].

Chitosan, a deacetylated derivative of chitin, presents a promising alternative due to its non-toxicity, biodegradability, natural abundance, and high density of amine and hydroxyl functional groups, which facilitate Cr(VI) binding [16,17,18,19]. However, its practical application is limited by poor stability under acidic conditions and low mechanical strength [20,21]. Consequently, chitosan modification strategies—such as crosslinking with glutaraldehyde, polyacrylamide, epichlorohydrin, or N-N′-ethylenediamine—have been widely investigated to enhance its physicochemical properties, stability, and reusability [22,23,24,25,26,27].

Quaternary ammonium-modified chitosan has shown particular promise in Cr(VI) removal. For instance, Sessarego et al. reported a phosphonium-crosslinked chitosan (PCC) synthesized using tetrakis hydroxymethyl phosphonium sulfate (THPS), achieving a Cr(VI) adsorption capacity of 93.0 mg/g [28]. However, the practical deployment of PCC is constrained by challenges in solid–liquid separation. Magnetic modification offers a potential solution by enabling facile recovery [29]. Notably, Fe-containing magnetic materials not only facilitate separation but also contribute to Cr(VI) reduction. For example, Li et al. developed a zirconium-doped carbon-coated magnetic Fe_3_O_4_ composite (Zr-Fe_3_O_4_@C) via a chitosan-assisted hydrothermal method, exhibiting high surface area (44.5 m^2^/g), strong magnetization (53.5 emu/g), and excellent dispersibility for Cr(VI) adsorption and reduction [30]. Additionally, Teng et al. demonstrated that chitosan coatings mitigate nanoparticle agglomeration, further facilitating Cr(VI) removal performance [31].

In this study, we synthesized a phosphorus-modified magnetic chitosan composite (PCC/Fe_3_O_4_) using chitosan as the matrix, Fe_3_O_4_ as the magnetic core, and THPS as the crosslinking agent. The composite was characterized by its structural and functional properties, and its Cr(VI) removal efficiency, regeneration capability, long-term stability, and magnetic separation performance were systematically evaluated through batch experiments.

## 2. Materials and Methods

### 2.1. Phosphorus-Modified Magnetic Chitosan (PCC/Fe_3_O_4_) Preparation

A 200.0 mL aqueous solution of 0.5% (*v*/*v*) acetic acid was prepared, into which 2.0 g of chitosan (CS, degree of deacetylation ≥ 95%, molecular weight range: 1.26 × 10^5^–2.65 × 10^5^ Da, Shanghai Macklin Biochemical Technology Co., Ltd., Shanghai, China) was dispersed. The mixture was magnetically stirred for 2 h at ambient temperature to achieve complete dissolution of chitosan. Under a nitrogen atmosphere to prevent oxidation, 1.0 g of Fe_3_O_4_ nanoparticles (average particle size < 50 nm, stated purity > 99%) was introduced into the chitosan-acetic acid solution. The suspension was subjected to ultrasonication for 1 h to ensure homogeneous dispersion and uniform coating of Fe_3_O_4_ nanoparticles with chitosan. Subsequently, 20 mL of tetrakis(hydroxymethyl)phosphonium sulfate (THPS, 75% aqueous solution) was added as a crosslinking agent. The reaction mixture was maintained at 70 °C in a water bath with continuous stirring at 320 rpm for 1 h to facilitate the formation of phosphonium-crosslinked chitosan (PCC) on the magnetic core. After cooling to room temperature for 1 h, the product was isolated by centrifugation at 3500 rpm for 10 min. The supernatant was decanted, and the residual solid was sequentially washed with deoxygenated water and ethanol to remove unreacted THPS and other impurities. The purified product was freeze-dried at −45 °C for 48 h, finely ground, and sieved through a 100-mesh sieve to obtain the final PCC/Fe_3_O_4_ composite.

### 2.2. Batch Experiments for Cr (VI) Removal

Batch experiments were systematically conducted at 25 °C to evaluate the influence of contact time and coexisting anions (CO_3_^2−^, SO_4_^2−^, NO_3_^−^, and Cl^−^, prepared with 0.5, 5, and 50 mmol/L sodium carbonate, sodium sulfate, sodium nitrate, and sodium chloride, respectively) on Cr(VI) removal efficiency by PCC/Fe_3_O_4_. A 250 mL aqueous K_2_Cr_2_O_7_ solution containing 1.00 mg/L Cr(VI) was adjusted to pH 6 with 0.1 M NaOH or HCl solution, and then mixed with 0.01 g of CS, PCC, Fe_3_O_4_, or PCC/Fe_3_O_4_ under controlled conditions. Herein, a concentration of 1 mg/L and pH 6 was used because it represents a ubiquitous contamination level and closely mimics real-world conditions [28]. The suspension was agitated at 180 rpm for predetermined intervals to ensure uniform interaction. After reaction, the mixture was filtered through a 0.45 μm membrane, and residual Cr(VI) concentrations were quantified via diphenylcarbazide spectrophotometry at 540 nm, a method validated for its specificity toward Cr(VI). The total Cr concentrations in the aqueous solution were also determined with an Optima 3000XL inductively coupled plasma-atomic emission spectrometer (ICP, Perkin Elmer, Waltham, MA, USA), and the Cr(III) concentrations were obtained from the difference between the total Cr and Cr(VI) concentrations. To ensure statistical reliability, duplicate experiments were performed, and the mean values were reported.

The Cr(VI) amounts q_e_ (mg/g) removed by CS, PCC, Fe_3_O_4_, or PCC/Fe_3_O_4_ are calculated from Equation (1), and the removal efficiencies are calculated from Equation (2):

q_e_ = V × (C_0_ − C_e_)/m (1)

removal efficiency = (C_0_ − C_e_)/C_0_ × 100% (2)

where C_0_ and Ce are the Cr(VI) initial and equilibrium concentration in solutions (mg/L), respectively, V is the volume of Cr(VI) solution (L), and m is the mass of the function materials used (g).

A quasi-first-order kinetic model [Equation (3)] and a quasi-second-order kinetic model [Equation (4)] were used to fit the kinetic data of Cr(VI) removal by CS, PCC and PCC/Fe_3_O_4_.

ln(q_e_ − q_t_) = lnq_e_ − k_1_t/2.303 (3)

t/q_t_ = 1/k_2_q_e_^2^ + t/q_e_ (4)

where q_t_ is the Cr(VI) removal capacity at time t, mg/g; k_1_ is the quasi-first-order kinetic constant, min^−1^; k_2_ is the quasi-second-order kinetic constant, g/(mg·min); t is the contact time, min.

### 2.3. Characterization of the PCC/Fe_3_O_4_

The phase composition of PCC/Fe_3_O_4_ was characterized by X-ray diffraction (XRD, Bruker D8 Advance, Karlsruhe, Germany) with a 2θ range of 10~80° at a scanning rate of 10°/min. Fourier-transform infrared spectroscopy (FT-IR, Thermo Nicolet iS5, Waltham, MA, USA) was employed to analyze chemical functional groups using KBr pellets, with spectra recorded from 400~4000 cm^−1^ at 4 cm^−1^ resolution (32 scans). Specific surface area and pore structure of CS, Fe_3_O_4_, PCC/Fe_3_O_4_ samples were determined via N_2_ adsorption–desorption isotherms (Quantachrome Nova 4000, Boynton Beach, FL, USA) at 77 K. Prior to analysis, the CS and PCC/Fe_3_O_4_ samples were degassed under vacuum at 60 °C for 10 h, respectively, while the bare Fe_3_O_4_ sample was degassed at 160 °C for 6 h. Magnetic properties were evaluated by vibrating sample magnetometry (VSM, Lake Shore 8604, Westerville, OH, USA) under applied fields of ±2 T at 25 °C. Surface morphology and elemental distribution were examined by scanning electron microscopy with energy-dispersive X-ray spectroscopy (SEM-EDS, ThermoFisher Apreo 2S+, Oxford UltimMax 65, High Wycombe, UK) at 5.0 kV after gold sputtering. Chemical states and atomic configurations were probed via X-ray photoelectron spectroscopy (XPS, Thermo Scientific Nexsa, Waltham, MA, USA), with data processed using Avantage 6.8.0 software for peak deconvolution and binding energy analysis.

### 2.4. Stability and Regeneration Test of PCC/Fe_3_O_4_

The stability of PCC/Fe_3_O_4_ was systematically evaluated by subjecting the prepared composite to accelerated aging under ambient conditions (25 °C, 60% relative humidity in air) for 1, 3, 5, and 25 d in an open container. Following aging, Cr(VI) removal experiments were conducted using the aged PCC/Fe_3_O_4_ under identical conditions as described in Section 2.1 (e.g., pH 6, 1.00 mg/L Cr(VI), 180 rpm agitation). The temporal evolution of Cr(VI) removal efficiency was quantitatively assessed to determine the composite’s degradation kinetics and long-term performance retention. Comparative analysis of removal efficiencies across aging intervals elucidated the correlation between material stability and functional durability, with particular attention to potential oxidation of Fe(II) active sites or chitosan matrix degradation.

The PCC/Fe_3_O_4_ samples after Cr(VI) adsorption were collected and oven-dried at 50 °C for 24 h to remove residual moisture. To assess their reusability, 0.01 g of Cr(VI)-saturated PCC/Fe_3_O_4_ was immersed in 10 mL of 0.5 mol/L H_2_SO_4_ elution solution under continuous agitation for 60 min to facilitate the desorption of Cr(VI) through protonation of active sites and dissolution of surface complexes. The regenerated PCC/Fe_3_O_4_ was subsequently rinsed with deionized water until neutral pH and reused for additional adsorption cycles to evaluate performance retention.

## 3. Results and Discussion

### 3.1. Characterization

**Figure 1:**
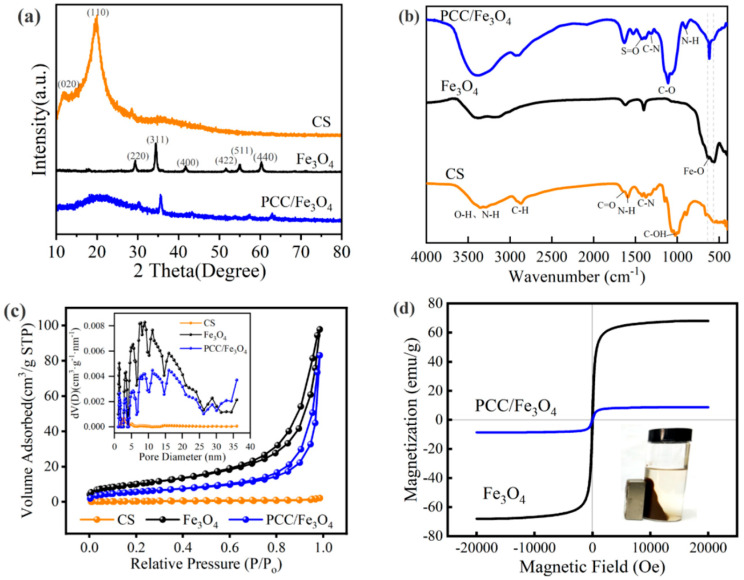
(**a**) XRD, (**b**) FT-IR spectra, (**c**) N_2_ adsorption–desorption isotherms and pore size distribution plots (insets) of CS, Fe_3_O_4_, and (**d**) Hysteresis loops of Fe_3_O_4_ and PCC/Fe_3_O_4_.

The XRD patterns of raw chitosan (CS), Fe_3_O_4_, and the as-synthesized PCC/Fe_3_O_4_ composite are presented in Figure 1a. Comparative analysis reveals that the unmodified CS exhibits two distinct characteristic diffraction peaks at 2θ = 10.4° and 20.0°, corresponding to the (020) and (110) crystal planes, respectively. The presence of abundant functional groups such as –OH and –NH_2_ facilitates the formation of extensive intermolecular and intramolecular hydrogen bonds. This hydrogen-bonding network promotes the molecular ordering that gives rise to the observed crystalline diffraction peaks [32]. After modification, the PCC/Fe_3_O_4_ composite exhibits diffraction peaks consistent with those of pure Fe_3_O_4_, observed at 2θ values of 30.1°, 35.5°, 43.1°, 53.4°, 57.0°, and 62.6°, which are indexed to the (220), (311), (400), (422), (511), and (440) crystal planes, respectively [33,34]. This indicates that the crystal structure of Fe_3_O_4_ remained intact during the synthesis of PCC/Fe_3_O_4_, although slight variations in peak intensity are noted. The broadened diffraction peaks and the absence of sharp, high-intensity reflections in the PCC/Fe_3_O_4_ pattern suggest that the composite possesses a crystallinity intermediate between that of amorphous CS and highly crystalline Fe_3_O_4_, indicating a relatively low degree of crystalline order. In addition, the PCC/Fe_3_O_4_ peaks were observed to shift to a higher degree compared with that of pristine Fe_3_O_4_. This phenomenon can be attributed to strong interfacial interactions, most likely coordination between phosphorus-containing groups from THPS and surface iron atoms of Fe_3_O_4_. This coordination induces compressive strain on the nanocrystals, providing evidence of successful chemical integration—rather than simple physical mixing—between the magnetic core and the phosphorus-modified chitosan matrix.

The FTIR spectra of CS, Fe_3_O_4_, and PCC/Fe_3_O_4_ are presented in Figure 1b. The spectrum of CS exhibits a broad absorption band around 3370 cm^−1^, corresponding to the overlapping O–H and N–H stretching vibrations, indicative of extensive hydrogen bonding involving –OH and –NH_2_ groups. Peaks observed at 2921 cm^−1^ and 2862 cm^−1^ are assigned to the asymmetric and symmetric C–H stretching vibrations, respectively. The bands at 1650 cm^−1^, 1592 cm^−1^, and 1376 cm^−1^ are attributed to the amide I C=O stretching, N–H bending, and C–N stretching of the remaining acetamido groups, respectively. Additionally, the peak near 1030 cm^−1^ corresponds to C–OH stretching vibrations [35,36]. In the spectrum of PCC/Fe_3_O_4_, the broad absorption at 3370 cm^−1^, associated with O–H and N–H stretching, appears stronger and sharper compared to that of pure CS, suggesting extensive cross-linking between THPS molecules and CS via hydroxyl groups. The peak at 1410 cm^−1^ is assigned to S=O stretching vibrations, resulting from electrostatic interaction between protonated amino groups of CS and sulfate ions. The absorption at 1310 cm^−1^ corresponds to C–N stretching of secondary amines, confirming the cross-linking reaction between amino groups of CS and hydroxyl groups of THPS. The band at 1110 cm^−1^ is attributed to C–O stretching, while the peak at 916 cm^−1^ represents out-of-plane N–H bending. Characteristic absorptions in the range of 500–750 cm^−1^ are associated with Fe–O stretching vibrations [37]. The P–C vibration is likely overlapped with or concealed by the Fe–O band within this region [28]. In summary, these results confirm the successful incorporation of THPS and CS with Fe_3_O_4_, forming a phosphorus-modified magnetic chitosan composite.

The specific surface area, pore volume, and pore size distribution of CS, Fe_3_O_4_, and PCC/Fe_3_O_4_ are summarized in Table 1 and illustrated in Figure 1c. Based on the IUPAC classification, the PCC/Fe_3_O_4_ composite displays a type IV adsorption isotherm accompanied by an H3-type hysteresis loop, which is characteristic of mesoporous materials [38,39]. The Brunauer–Emmett–Teller (BET) surface area was determined to be 20.7 m^2^/g, with a total pore volume of 0.129 cm^3^/g and an average pore diameter of 24.9 nm, further corroborating the mesoporous structure of the synthesized PCC/Fe_3_O_4_ material.

The magnetic properties of Fe_3_O_4_ and PCC/Fe_3_O_4_ were characterized using vibrating sample magnetometry (VSM) at room temperature (Figure 1d). Both samples exhibited negligible coercivity and remanence, confirming their superparamagnetic nature. The saturation magnetization (M_S_) of PCC/Fe_3_O_4_ was measured to be 8.67 emu·g^−1^, significantly lower than that of unmodified Fe_3_O_4_ nanoparticles (68.0 emu·g^−1^). This reduction can be attributed to the diamagnetic contribution from the non-magnetic chitosan (CS) and THPS shell. Despite the decrease, PCC/Fe_3_O_4_ retained sufficient magnetic responsiveness to enable rapid solid–liquid separation under an external magnetic field, as illustrated schematically in Figure 1d.

### 3.2. Cr(VI) Removal of PCC/Fe_3_O_4_ and the Influencing Factors

**Figure 2:**
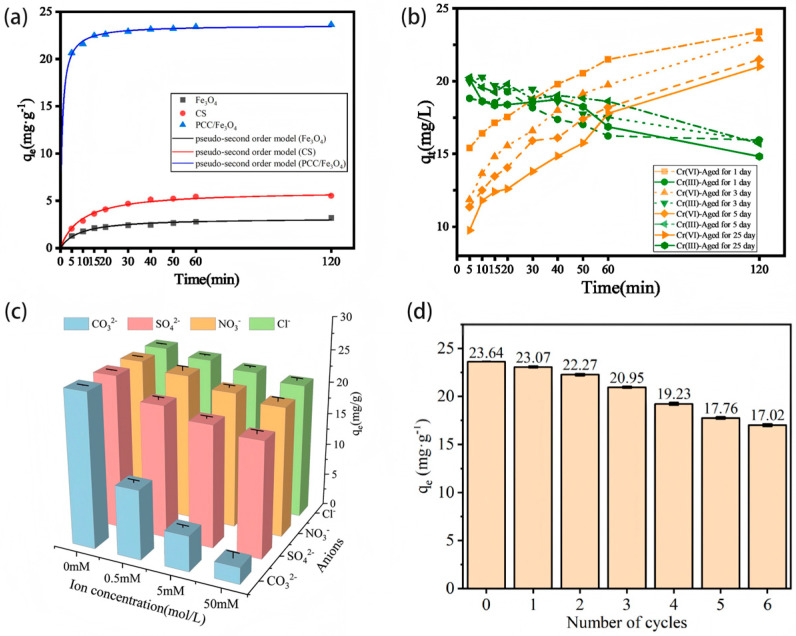
Cr(VI) removal by Fe_3_O_4_, CS and PCC/Fe_3_O_4_ (**a**) fitted with kinetic adsorption models, (**b**) influenced with aging time, (**c**) the coexisting anions, and (**d**) the recycled number.

As shown in Figure 2a, the Cr(VI) adsorption capacity (q_t_) of PCC/Fe_3_O_4_ reached 23.6 mg/g, which is notably higher than that of Fe_3_O_4_ (3.20 mg/g) and CS (5.52 mg/g). As benchmarked against previously reported adsorbents in Table 2, PCC/Fe_3_O_4_ exhibits a comparable Cr(VI) adsorption capacity, notably under a much lower initial concentration and more moderate conditions. Correspondingly, the Cr(VI) removal efficiency achieved by PCC/Fe_3_O_4_ was also substantially greater, demonstrating its superior performance for Cr(VI) remediation.

Kinetic analysis indicated that both models applied effectively described the adsorption process (R^2^ ≥ 0.995) [45]. The pseudo-second-order kinetic model exhibited a stronger correlation (as listed in Table 3), yielding a calculated q_e_; value of 23.6 mg/g that is consistent with the experimental result, suggesting that the adsorption follows pseudo-second-order kinetics and is predominantly controlled by chemisorption.

Figure 2c illustrates the influence of coexisting anions on Cr(VI) removal by PCC/Fe_3_O_4_. The inhibitory effects followed the order: CO_3_^2−^ > SO_4_^2−^ > NO_3_^−^ > Cl^−^. Increasing the CO_3_^2−^ concentration from 0 to 50 mmol L^−1^ resulted in a sharp decline in Cr(VI) removal efficiency from 94.5% to 10.1%, which is likely attributable to the accompanying rise in pH that inhibits Cr(VI) reduction [46]. Similarly, the presence of SO_4_^2−^ at 50 mmol L^−1^ reduced the removal efficiency to 70.9%. The pronounced competitive effect of SO_4_^2−^ can be explained by two main reasons: (1) the structural similarity between SO_4_^2−^ and HCrO_4_^−^, as both ions adopt tetrahedral geometries with comparable ionic radii (0.029–0.034 nm for S(VI) vs. 0.033–0.052 nm for Cr(VI)) [47], and (2) the higher charge density of SO_4_^2−^ relative to Cl^−^ and NO_3_^−^, causing stronger electrostatic attraction to the positively charged -NH_2_ groups on PCC/Fe_3_O_4_ [48].

Figure 2b shows the effect of aging time on the Cr(VI) removal efficiency of PCC/Fe_3_O_4_. Aging exhibited only a limited effect on the removal performance: after 2 h, the removal efficiency remained as high as 86% on day 5 and 84% on day 25, compared to that of the freshly prepared adsorbent. The slight decrease may be due to the gradual oxidation of Fe_3_O_4_ upon prolonged exposure to air [49]. Chitosan, serving as both a coating and a support within the composite, plays a dual role: (1) increasing the specific surface area and active sites of the adsorbent, and (2) protecting Fe_3_O_4_ from O_2_, thus reducing its oxidation [50]. This protective effect considerably enhances the antioxidant stability of the material, prolonging its storage lifetime and maintaining its Cr(VI) removal capacity.

Figure 2d presents the regeneration performance of PCC/Fe_3_O_4_. The Cr(VI) removal capacity exhibited a gradual decline with increasing regeneration cycles, likely due to incomplete desorption of adsorbed Cr(VI) and partial loss of active sites. After the first cycle, the Cr(VI) removal capacity remained at 23.07 mg/g, retaining 97.6% of its initial efficiency. Even after six adsorption–desorption cycles, PCC/Fe_3_O_4_ maintained a 71.7% removal efficiency, demonstrating its excellent reusability for Cr(VI) removal. Future work should include more stability studies to further the evaluation of the long-term Cr(VI) removal performance of the regenerated PCC/Fe_3_O_4_.

### 3.3. Adsorption Mechanism

**Figure 3:**
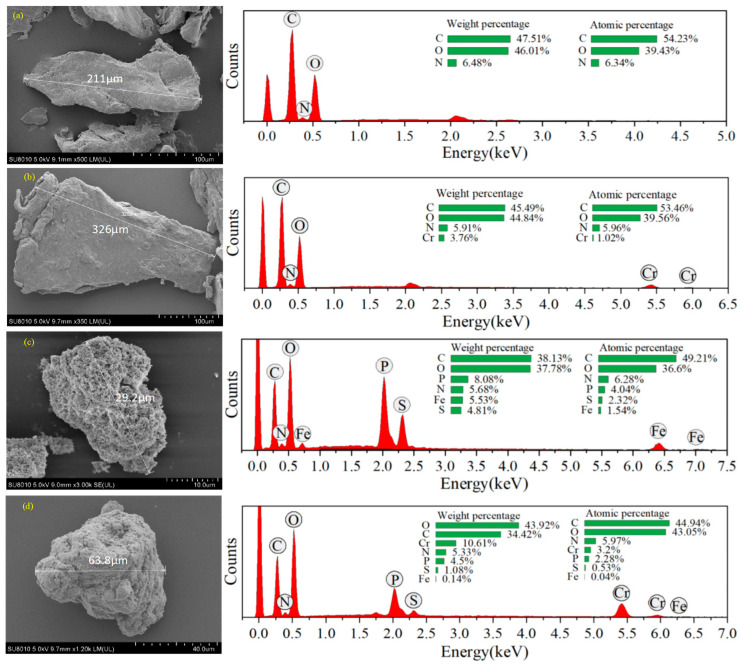
SEM-EDX of (**a**) chitosan, (**b**) chitosan with adsorbed Cr(VI), (**c**) PCC/Fe_3_O_4_, and (**d**) PCC/Fe_3_O_4_ with adsorbed Cr(VI).

The morphological and elemental characteristics of CS and PCC/Fe_3_O_4_ before and after Cr(VI) removal were examined using SEM-EDS, as presented in Figure 3. The PCC/Fe_3_O_4_ composite reveals a well-defined core–shell structure [51], characterized by a porous internal architecture and a rough surface morphology (Figure 3c). In contrast, pristine CS exhibits a smooth, sheet-like appearance with a dense surface [52]. EDS analysis confirmed the presence of C, N, O, P, S, and Fe on the surface of PCC/Fe_3_O_4_, with weight percentages of 38.13%, 5.68%, 37.78%, 8.08%, 4.81%, and 5.53%, respectively.

The EDS spectra further revealed the presence of Cr (10.6%), providing direct evidence of successful Cr(VI) uptake (Figure 3d). That EDS spectrum also revealed a sharp decrease in iron content (from 5.53% to 0.14%), which can be attributed to the reductive dissolution of Fe_3_O_4_. This process was initiated by the oxidation of structural Fe(II) by Cr(VI), leading to the disruption of the crystal lattice and subsequent dissolution of iron into the solution. Consequently, the concomitant loss of S and P is explained by the release of THPS-derived components upon the disintegration of the magnetic core.

**Figure 4:**
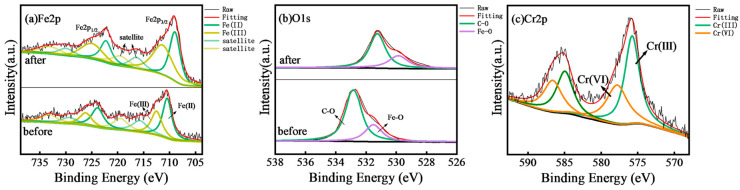
XPS Spectra of PCC/Fe_3_O_4_ adsorbent before and after Cr(VI) removal: (**a**) Fe2p, (**b**) O1s and (**c**) Cr2p.

X-ray photoelectron spectroscopy (XPS) was employed to elucidate the Cr(VI) removal mechanism by PCC/Fe_3_O_4_. The Fe2p spectrum (Figure 4a) exhibited peaks at 724.5 eV (Fe2p_1_/_2_) and 710.8 eV (Fe2p_3_/_2_), corresponding to Fe(II) (710.5 eV, 723.9 eV) and Fe(III) (712.6 eV, 726.2 eV) species [53,54,55]. Post-adsorption, these peaks shifted to lower binding energies (Fe(II): 709.0 eV, 722.3 eV; Fe(III): 711.5 eV, 725.2 eV), accompanied by a reduction in Fe(II) content (54.6% → 49.1%) and an increase in Fe(III) (45.4% → 50.9%). This confirms the oxidation of Fe(II) by Cr(VI), leading to Fe(III) and Cr(III) chemosorption on the adsorbent surface. The O1s spectrum (Figure 4b) revealed peaks at 532.7 eV (C-O) and 530.9 eV (Fe-O) [56,57], which shifted to 531.2 eV and 529.4 eV after adsorption, indicating hydrogen bond formation during Cr(VI) uptake [58,59]. The Cr2p spectrum (Figure 4c) displayed dual peaks at 585.5 eV (Cr2p_1_/_2_) and 576.2 eV (Cr2p_3_/_2_), deconvoluted into Cr(III) (575.9 eV, 585.0 eV) and Cr(VI) (577.5 eV, 586.3 eV) [30,60]. Quantitative analysis showed 61.6% of adsorbed chromium existed as Cr(III), further corroborating the redox reaction between PCC/Fe_3_O_4_ and Cr(VI). The XPS results demonstrate a synergistic mechanism involving (1) Fe(II)/Fe(III) redox cycling, (2) hydrogen bonding via oxygen functional groups, and (3) Cr(VI) reduction to less toxic Cr(III), followed by coprecipitation. This aligns with findings from similar Fe_3_O_4_-based composites.

**Figure 5:**
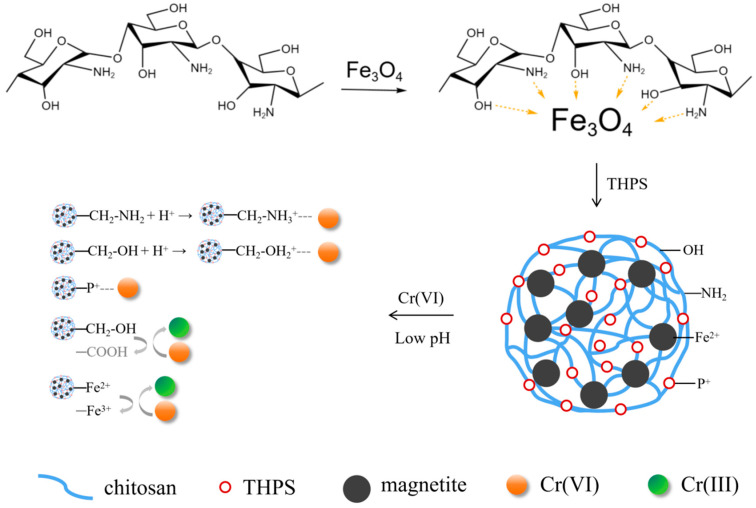
Proposed mechanisms of Cr(VI) removal by PCC/Fe_3_O_4_.

Based on the above analysis, a possible mechanism for removing Cr(VI) by PCC/Fe_3_O_4_ was proposed (Figure 5): After chitosan is modified by tetrakis hydroxymethyl phosphonium sulfate, PCC/Fe_3_O_4_ contains a large number of phosphating functional groups (quaternary phosphorus groups) [28,61], as well as groups such as -OH, and -NH_2_ [57]. Cr(VI) exists as pH-dependent anions (Cr_2_O_7_^2−^, HCrO_4_^−^, CrO_4_^2−^), with HCrO_4_^−^ dominating under acidic conditions (pH 2–6) [6], so the Cr(VI) removal mechanism by PCC/Fe_3_O_4_ integrates (1) electrostatic adsorption, (2) redox-driven reduction, and (3) coprecipitation, leveraging the synergistic effects of phosphating groups, protonated moieties, and Fe_3_O_4_’s redox activity.

Under acidic conditions, quaternary phosphorus groups and protonated –OH_2_^+^/–NH_3_^+^ on PCC/Fe_3_O_4_ provide strong electrostatic attraction for Cr(VI) anions (e.g., HCrO_4_^−^) [Equations (5) and (6)]:

R-CH_2_-NH_2_ + H^+^ → R-CH_2_-NH_3_^+^ (5)

R-CH_2_-OH + H^+^→ R-CH_2_-OH_2_^+^ (6)

Anions containing Cr(VI) are immobilized on the PCC/Fe_3_O_4_ material through electrostatic attraction. Subsequently, the –OH of PCC/Fe_3_O_4_ is gradually oxidized to –COOH in an acidic Cr(VI) aqueous solution, and free electrons are generated [Equations (7) and (8)].

R-CH_2_-OH → R-CHO + 2H^+^ + 2e^−^ (7)

R-CHO + H_2_O → R-COOH + 2H^+^ + 2e^−^ (8)

In a weak acid environment, due to the hydrolysis of dichromate, the dynamic equilibrium of three anions, Cr_2_O_7_^2−^, HCrO_4_^−^, and CrO_4_^2−^, may exist in solutions [62] [Equation (9)]. Through the action of Fe^2+^, Cr(VI) obtains free electrons in Equations (7) and (8) and is reduced to Cr(III) [Equations (10)–(12)]. Most of the Cr(III) ions produced are immobilized on the PCC/Fe_3_O_4_ surface, and only a small number of ions will dissolve in the solution.

2CrO_4_^2−^ + 2H^+^ ⇌ 2HCrO_4_^−^ ⇌ Cr_2_O_7_^2−^ + H_2_O (9)

HCrO_4_^−^ + 3Fe^2+^ + 7H^+^ → Cr^3+^ + 3Fe^3+^ + 4H_2_O (10)

CrO_4_^2−^ + 3Fe^2+^ + 8H^+^ → Cr^3+^ + 3Fe^3+^ + 4H_2_O (11)

(1 − x)Fe^3+^ + xCr^3+^ + 3H_2_O → Cr_x_Fe_1−x_ (OH)_3_ + 3H^+^ (12)

## 4. Conclusions

The efficacy of traditional Fe-based materials for remediating Cr(VI)-contaminated environments is constrained by inherent limitations, including low reactive surface area, propensity for oxidative passivation, and particle aggregation. Although chitosan-based coatings have been applied to enhance the stability and reactivity of Fe-based materials, they often impede efficient solid–liquid separation, complicating the recovery and reuse of spent adsorbents. To address these challenges, a novel phosphorus-modified magnetic chitosan composite (PCC/Fe_3_O_4_) was synthesized in this study using Fe_3_O_4_ as the magnetic core and tetrakis(hydroxymethyl)phosphonium sulfate (THPS) as a cross-linking agent. Comprehensive characterization via XRD, FTIR, BET, VSM, SEM, and XPS confirmed the successful incorporation of phosphorous functional groups (e.g., quaternary phosphonium) and chitosan reactive moieties (–OH, –NH_2_) onto the Fe_3_O_4_ surface, forming a stable composite structure. The material exhibited a high specific surface area of 20.67 m^2^ g^−1^ and superparamagnetic behavior, enabling efficient adsorption and facile magnetic separation.

PCC/Fe_3_O_4_ demonstrated superior Cr(VI) removal performance compared to unmodified chitosan and bare Fe_3_O_4_, achieving a maximum adsorption capacity of 23.6 mg g^−1^ under selected conditions (pH 6, initial Cr(VI) concentration = 1 mg L^−1^). Adsorption kinetics followed the pseudo-second-order model, and the removal mechanism involved: (i) electrostatic attraction between protonated –NH_3_^+^/–OH_2_^+^ groups and Cr(VI) anions under acidic conditions; (ii) redox reaction facilitated by Fe(II)/Fe(III) cycling; (iii) hydrogen bonding; and (iv) ligand exchange with phosphorus groups, enhancing Cr(III) immobilization. The adsorbent showed minimal interference from common anions such as NO_3_^−^ and Cl^−^, while CO_3_^2−^ and SO_4_^2−^ exhibited moderate inhibitory effects due to stronger competition with Cr(VI) oxyanions (e.g., HCrO_4_^−^). After aging for 5 days, PCC/Fe_3_O_4_ retained 86% of its initial Cr(VI) removal efficiency and maintained over 90% removal after five consecutive adsorption–desorption cycles, underscoring its robust cross-linked structure and excellent reusability.

PCC/Fe_3_O_4_ combines high adsorption capacity, exceptional recyclability, and convenient magnetic separation, offering a sustainable and efficient solution for the treatment of chromium-laden wastewater. Its performance surpasses that of conventional chitosan-based adsorbents and shows strong potential for practical application in cost-effective environmental remediation.

## Figures and Tables

**Table 1:** BET analysis of adsorbent materials.

Adsorbent Material	Specific Surface Area (m^2^/g)	Average Pore Size (nm)	Total Adsorption Pore Volume (cm^3^/g)
CS	1.058	12.30	0.0033
Fe_3_O_4_	36.502	16.58	0.1513
PCC/Fe_3_O_4_	20.671	24.88	0.1286

**Table 2:** Comparison of various adsorbents for Cr(VI) removal.

Adsorbent Material	Adsorption Conditions				Adsorption Capacity (mg/g)	Ref.
	Initial Conc (mg/L)	Temp. (°C)	Time (min)	pH		
Chitosan-coated sour cherry kernel shell beads	10.0	25	45	2	24.5	[40]
Poly-sulfone/diethylenetriaminepentaacetic acid-chitosan composite beads	120	28	1440	5	56.72	[41]
Magnetic chitosan graphene oxide composite	40	25	180	2	100.51	[42]
Sodium alginate-polyvinyl alcohol-chitosan composite	10.0	35	60	2	37.7	[43]
Banana trunk fiber-reinforced chitosan	50.0	27	120	7	12.7	[44]
PCC/Fe_3_O_4_	1.00	25	60	6	23.6	This study

**Table 3:** Cr(VI) kinetic adsorption by CS and PCC/Fe_3_O_4_ fit with dynamic models.

Adsorbents	Quasi-First-Order Dynamic Model			Pseudo-Second Order Model		
	k_1_/min^−1^	R^2^	q_e_/mg·g^−1^	k_2_/min^−1^	R^2^	q_e_/mg·g^−1^
Fe_3_O_4_	0.096	0.950	2.759	0.037	0.985	3.187
CS	0.076	0.993	5.398	0.017	0.976	6.066
PCC/Fe_3_O_4_	0.441	0.995	22.931	0.055	0.999	23.576

## Data Availability

The original contributions presented in this study are included in the article. Further inquiries can be directed to the corresponding author.
